# Variable renewable energy penetration impact on productivity: A case study of poultry farming

**DOI:** 10.1371/journal.pone.0286242

**Published:** 2023-10-02

**Authors:** Marie-Cécile Dupas, Sophie Parison, Vincent Noel, Petros Chatzimpiros, Éric Herbert

**Affiliations:** 1 Université Paris Cité, CNRS, UMR 8236 - LIED, Paris, France; 2 CNRS, Université de Toulouse, UMR 8539 - LAERO, Observatoire Midi-Pyrénées, Toulouse, France; Southwest Jiaotong University, CHINA

## Abstract

Like all current industrial systems, agriculture overwhelmingly relies on energy supply from controllable sources, mainly fossil fuels and grid electricity. Power supply from these sources can be adapted to perfectly match the timing of power requirements of demand systems. The energy transition largely consists in substituting renewable power—which is intermittent by nature—to controllable sources, leading to disconnection between instantaneous power production and demand. Energy storage is a potential solution for balancing production and demand and safeguarding the operating conditions of the demand system. In this paper we quantify the effects of renewable power supply (solar and wind) on the operation of a standard poultry farm. We model the balance of power generation and demand considering the growth conditions of poultry and local weather data including temperatures, wind speed and solar radiation. We assess scenarios of renewable power supply in function of the size of the power plant, the wind-to-solar power generation mix and energy storage, and assess the impact of power supply patterns on the operating intensity (productivity) of the demand system. We show that, with a limited storage capacity, it is possible to achieve non-negligible shares of renewable power penetration without major loss in farm productivity. However, a full transition to renewable power would require the combination of *i*)-large energy storage compared to the annual demand, *ii*)- significant oversizing of the power production plant, and *iii*)-the exclusion of power generation combinations (wind/solar) that deviate from the timing of demand. Storage and power plant oversizing is all the more critical as production and demand are uncorrelated over the year. The ratio of useful to unused energy storage by the end of the year varies with the energy mix and operating intensity (productivity) of the farm. We discuss the implications of different energy configurations on the performance of the demand system.

## Introduction

Food systems today overwhelmingly rely on energy inputs from non-renewable energy stocks, mainly fossil fuels [1, 2]. Energy stocks are controllable, in that they can supply a system according to the timing of the system’s needs [3, 4]. In contrast to energy stocks, Variable Renewable Energy flows—called VRE hereafter—such as wind and solar, are intermittent by nature and, thereby, out-of-phase compared to the demand of a given system. VRE is function of seasons, climate and weather factors, and translates into variable electricity loads in electric grids which are difficult to manage from a grid operation perspective [5]. A solution to tackle this variability is energy storage allowing for asynchronous penetration of VRE in the energy mix (see *e.g.* [6, 7] for a review).

The key role of energy in agricultural production, and, in particular, the heavy dependence of industrial farming systems on fossil fuels [2, 8] is a major sustainability issue and implies that the energy transition in the agricultural sector is a great challenge. Today, the agricultural sector relies little on electricity because most of its energy requirements come from mobile machinery of high nominal power which is difficult to electrify with current energy densities of batteries [9]. Electricity is mostly used in on-grid facilities such as agricultural buildings and stationary devices, and its share in total energy may greatly vary between crop and livestock oriented farms. Power demand from livestock farms has been dramatically increasing for decades in many countries as a result of the intensification and automation of the livestock sector [10, 11]. The dominant energy use in livestock units is for heating and ventilation of buildings. In the case of the French poultry sector, heating and ventilation are estimated at 1.81 TWh/year at the national scale [12], which is about 70% of total current annual energy consumption (excluding feed) of the poultry sector [13].

Within the context of climate change, decarbonation of the energy mix concerns all sectors and is being given increasing attention in the scientific literature [14, 15]. Wind and solar electricity generation is diffusing rapidly [16] and is typically injected into large-scale interconnected grids [17]. With increasing penetration of intermittent sources, the storage requirements of electric systems also increase, and transition scenarios typically assess production and storage requirements at aggregate national and continental scales [7, 18–22]. In contrast, little emphasis is put on the effect of VRE penetration at smaller scales, which is relevant in assessing the criticality of energy storage on specific sectors. Several works highlight the need for rapid, low-volume storage that can be decentralized—*e.g.* [23] report a gravity solution that can be implemented in buildings—but, to the best of our knowledge, there is no systematic analysis on particular sectors. Potential flexibility in electricity demand by taking advantage of thermal inertia or flexibility in activating a machine may also help increasing direct VRE penetration—and decreasing storage requirements—although such options are barely explored in the scientific literature.

In this paper, we develop a semi-empirical modelling approach of a decentralized production system, *i.e.* a poultry building, for which we consider the heating and ventilation requirements that we supply with VRE (wind and solar) input. The paper provides insights on the dynamics between intermittent energy supply, energy storage and potential degradation of the operating conditions of the demand system. The system power balance, including power requirements and generation, is largely dictated by the seasonal cycles and weather conditions. The power requirement is largely dictated by the temperature gap between the target inside air temperature which is specific to the birds’ metabolic needs, and the outside air temperature, as well as it includes a ventilation requirement which is also function of temperature. Deficient power supply compared to demand, impedes respecting the target air temperature, and has a direct impact on the functioning of the system, in particular by reducing its operation intensity. The operation intensity is defined as the annual duration for which target temperature and ventilation are respected.

In summary, our modeling approach allows assessing *i*)-the effect of intermittent wind and solar power mix combinations and sizing on total energy production and use by a decentralized demand system, *ii*)-the effect of energy storage on improving VRE penetration and supporting the satisfaction of the demand system’s requirements and operating intensity.

The structure of the paper is as follows. *Material and Methods* provide the description of the model, including energy balance equations between demand, supply and storage, the model parameters and assumptions used and the scenarios explored. The scenarios differ in terms of energy mix, storage and sizing of the renewable power production plant. *Results* provide insights on the effect of the energy supply regime and storage capacity on the demand system using the inside farm air temperature *T*_in_ as a proxy of the farm operating intensity *τ*. *Discussion* examines trade-offs between the energy mix, storage requirements and the satisfaction of the power needs of the demand system, and puts the findings in perspective with energy transition analysis at larger spatial scales. We conclude with a brief summary and generalization of the key insights of the study.

## Materials and methods

**Fig 1:**
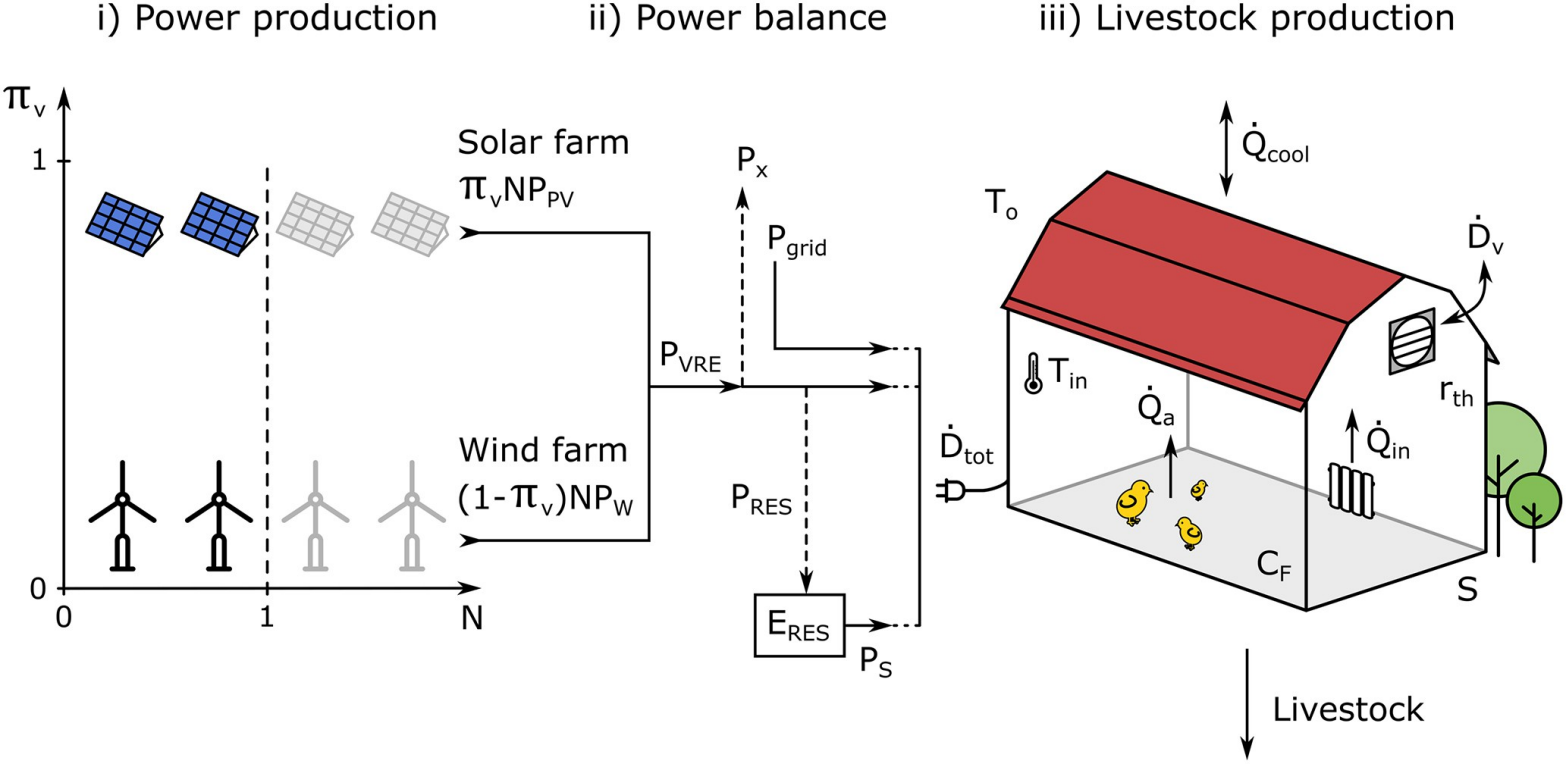
Global structure of the modelling. **i) Power production** is sized considering instantaneous photovoltaic (*P*_PV_) and wind (*P*_W_) power generation, energy mix between solar and wind power (*π*_*v*_) and a dimensioning factor (*N*) of the power plant. For *N* > 1 annual renewable energy production exceeds annual demand and for *N* < 1 annual renewable energy production is below annual demand. *π*_*v*_ is comprised between 0 (only wind) and 1 (only PV). *P*_VRE_ is total instantaneous renewable power production, see Eq 4. **ii) The power balance** connects power demand (${\dot{D}}_{\text{tot}}$) of the farm to power supply considering direct penetration of renewable power (*P*_VRE_) and power delivered either from storage (*P*_*S*_) or the grid (*P*_grid_). If *P*_VRE_ exceeds instantaneous power demand, excess instantaneous power is either stored (*P*_RES_) or exported to the grid (*P*_x_) depending on the scenario (see Table 4). Renewable energy storage (*E*_RES_) is ∫(*P*_RES_ − *P*_*S*_)d*t*. **iii) The enthalpic balance of the poultry production unit** is calculated as the sum of power required for heating (${\dot{Q}}_{\text{in}}$) and ventilating (${\dot{D}}_{\text{v}}$) the building, plus enthalpy generated by the animals (${\dot{Q}}_{\text{a}}$) minus heat exchange between the building and the atmosphere (${\dot{Q}}_{\text{cool}}$) through advection (ventilation) and convection. *T*_in_ and *T*_o_ are respectively inside and outside temperatures. *C*_*F*_ is the heat capacity of the total volume of the farm (air and envelope) and *r*_*th*_ is the thermal resistance of the envelope. *S* is the ground area of the farm. Table 1 summarizes the numerical values used in the simulations.

The poultry farm in our study is composed by three interacting components: (*i*)-energy generation from wind (W) and photovoltaic (PV) power plants, (*ii*)-energy storage, and (*iii*)-energy demand for heating and ventilation of the poultry building. The global structure of the modeling is shown in Fig 1. The following subsections describe the numerical parameters and input data used in the modeling. All parameters and variables are summarized in Table 1. Table 2 summarizes the main parameters. For the rest of this paper, extensive quantities related to the energy balance are supposed normalized by *S* the farm’s surface.

**Table 1:** Input and output parameters of the model. Quantities with ⋆ are normalized by *E*_ref_. VRE is the Variable Renewable Energy.

**Input parameters**			
Poultry farm building			
*r*_*th*_	Thermal resistance of the envelope	1.9	K/W/m²
*c*_*g*_	Heat capacity of air	1.12	kJ/m^3^/K
*c*_*i*_	Heat capacity of insulation	97.2	kJ/m^3^/K
*c*_*c*_	Heat capacity of concrete	2.41	MJ/m^3^/K
*C*_*F*_	Farm total heat capacity	198	MJ/K
*h*	Height	2.6	m
*ℓ*	Width	10	m
*L*	Length	40	m
*S*	Ground surface	400	m²
*S*_*out*_	External surface of the envelope	660	m²
Animals (chicks)			
*T*_T_	Inside target temperature	32	°C
*δT*	Working temperature range	±1	°C
${\dot{Q}}_{a}$	Sensible heat of the animals	10.8	kW
${\dot{q}}_{a}$	Sensible heat per unit of mass	9	W/kg
${\dot{Q}}_{in}$	Heating power	0−32	kW
${\left({\dot{Q}}_{in}\right)}_{max}$	Maximum heating power	80	W/m²
*ρ*	Number of chicks per unit surface	30	1/m²
*m*_*a*_	Mass of one chick	0.1	kg
${\dot{d}}_{m}$	Ventilation rates required for chicks	0.8−5	m^3^/h/kg
*D*	Animal growth time duration	1	hour
Weather			
*T*_o_	Outside air temperature, 2 m high		°C
*v*	Wind speed, 80 m high		m/s
*ψ*	Solar radiation		W/m²
*y*	Year (Sept. 1st 2013−Aug. 31th 2014)	0−1	−
*t*	Time		s
*δt*	Iteration time step	60	s
Sizing of the power production units			
*B*_PV_	Solar power plant sizing	0.52	m_PV^2^_/m²
*B*_W_	Wind power plant sizing	4.78	m_W^2^_/m²
*π*_*v*_	Solar fraction in energy mix	0−1	−
*N*	Power plant dimensioning factor	0−2	−
*E*_ref_	Ideal annual energy consumption	85	kWh/m²
*E*_VRE_	VRE annual production	⋆	−
*E*_grid_	Energy from the grid	⋆	−
*E*_tot_	Total energy (*E*_VRE_ + *E*_grid_)	⋆	−
**Output parameters**			
Enthalpic balance and power demand			
*T*_in_	Inside air temperature		°C
*Q*_*B*_	Enthalpy of stock in the building		J
${\dot{Q}}_{\text{cool}}$	Cooling enthalpy outflow rate		W
${\dot{D}}_{V}$	Ventilation power demand	0.24−1.52	kW
${\dot{d}}_{V}$	Ventilation power per unit surface	0.6−3.8	W/m²
${\dot{D}}_{tot}$	Total instantaneous power demand		W
Power balance			
*P*_VRE_	Renewable power production		W
*P*_PV_	Solar PV electrical power		W
*P*_W_	Wind turbine electrical power		W
*P*_S_	Outgoing renewable storage power		W
*P*_x_	Excess power exported		W
*P*_RES_	Excess power stored		W
*E*_RES_	Renewable energy in storage	⋆	−
*E*_S_	Energy in storage (*E*_RES_ + *E*_grid_)	⋆	−
*E*_req_	Required storage sizing	⋆	−
Livestock production			
*τ*	Farm operating time ratio	0−1	

**Table 2:** Summary of the main modelling parameters. *T*_T_, *m*_*a*_, ${\dot{q}}_{a}$ and *ρ* are, respectively, the target inside air temperature, the mass of one animal, the sensible heat flow per animal mass unit and the density of animals in the farm (values are from [24]). *L*, *ℓ*, *h*, ${\left({\dot{Q}}_{in}\right)}_{max}$, ${\dot{d}}_{V}$ and *C*_*F*_ are respectively the farm’s length, width and height, the maximum installed power for heating, the ventilation power rate and total thermal capacity, see Eq 2, of the farm. The last column corresponds to reference outputs in standard conditions that are necessary to parameterize power supply simulations. *E*_ref_ is the farm’s standard annual energy requirement per unit area, computed from weather data of outside air temperature, see Fig 2. *B*_PV_ and *B*_W_ are scale factors respectively for PV and wind power allowing evaluating the ground surface needed to produce *E*_ref_. *H*/*E*_ref_ is the fraction of energy dedicated to heating over the year.

Animals		Farm parameters		Energy	
*T*_T_ [°C]	32 ± 1	*L* × *ℓ* ×*h* [m^3^]	40 × 10 × 2.6	*E*_ref_ [kWh/m^2^]	85
*m*_*a*_ [kg]	0.1	${\left({\dot{Q}}_{in}\right)}_{max}$ [W/m^2^]	80	*B*_PV_ [m_PV^2^_/m²]	0.52
${\dot{q}}_{a}$ [W/kg]	9	${\dot{d}}_{V}$ [W/m^2^]	0.6–3.8	*B*_W_ [m_W^2^_/m²]	4.78
*ρ* [1/m^2^]	30	*C*_*F*_ [MJ/K]	198	*H*/*E*_ref_ [—]	0.70

### Parameters of the modelled farm

#### Enthalpic balance of the poultry building

The poultry building is described by the following enthalpic balance:

$$\begin{array}{cc} \frac{\partial Q_{B}}{\partial t}+{\dot{Q}}_{\text{cool}} & ={\dot{Q}}_{\text{in}}+\dot{Q_{a}} \end{array}$$ (1)

Solar radiation directly hitting the farm is not considered. ${\dot{Q}}_{\text{cool}}$ is the transport of sensible enthalpy by conduction (thermal leakage) and advection (ventilation). The enthalpic balance per unit area is written ${\dot{Q}}_{\text{cool}}=\alpha \;\Delta T/S$ with *α* = *c*_*g*_*w* + *S*_out_/*r*_*th*_ the sum of the leakage terms. *c*_*g*_*w* is the contribution of ventilation, *c*_*g*_ is air heat capacity at constant pressure per unit of volume, and *w* the flow rate (m^3^/s). The conductive contribution is *S*_out_/*r*_*th*_, *r*_*th*_ being the thermal resistance of the farming building and *S*_out_ = *S* + 2*h*(*L* + *ℓ*) the external surface, with *S* the ground area, *h* the height, *L* and *ℓ* the width and depth respectively. The numeric values are given in Table 1. $\dot{Q_{a}}$ is the sensible enthalpy provided by animals, ${\dot{Q}}_{\text{in}}$ is that provided by heating, and *Q*_*B*_ is the trade-off enthalpy of the building. $\frac{\partial Q_{B}}{\partial t}$ is null at constant temperature. Inside temperature (*T*_in_) and *Q*_*B*_ are related through the total thermal capacity of the farm *C*_*F*_ in Eq 2,

$$\begin{array}{cc} \frac{\partial Q_{B}}{\partial t} & =C_{F}\;\frac{dT_{\text{in}}}{dt} \end{array}$$ (2)

#### Farm parameters adjusted on realistic values

The agricultural production unit is defined according to the criteria described hereafter and observed in existing production systems [13]. We consider an intensive poultry production unit with high animal density per unit area, denoted *ρ*. Typical animal density is about *ρ* = 15 m^−2^ for chicken and *ρ* = 30 m^−2^ for chicks. We focus on chicks because their growth conditions are the most constraining in terms of inside air temperature (*T*_T_) and ventilation (${\dot{d}}_{v}$) translating into higher energy requirements.

The modelling considers a typical farm building made of concrete with thermal insulation for walls, roof and the ground (see Table 1 for values of thermal parameters). Walls thickness is adjusted so that average thermal resistance of the farm’s envelope is *r*_*th*_ = 1.9K/W/m^2^, as proposed in [13, 25] which provides thermal conductance standard values for walls, ground and rooftop of such facilities. The ground surface and height of the building are respectively *S* = 400 m^2^ and 2.6 m, corresponding to total volumetric heat capacity of *C*_*F*_ = 198MJ/K.

#### Heating

We consider a chick production unit with average animal density. A chick grows over six weeks on average. The recommended inside air temperature for the first weeks is *T*_T_ = 32°C [13]. We define the interval 31–33°C as the inside target air temperature for chicks growth.

According to [24], the sensible heat flow per unit mass of chick is ${\dot{q}}_{a}=9\;$W/kg, that is ${\dot{Q}}_{a}=\rho \;S\;m_{a}\;\dot{q_{a}}=27\;$ W/m^2^ considering average chick mass of *m*_*a*_ = 0.1kg and *ρ* = 30 chicks/m^2^. This corresponds to heat flow of $\dot{q_{a}}=10.8$ kW and annual heat dissipation inside the building of 234 kWh/m^2^.

The maximum heating capacity of the building is set to ${\left({\dot{Q}}_{\text{in}}\right)}_{\text{max}}∼80\;$ W/m^2^ based on [12], which is roughly three times higher than the continuous sensible heat flow generated by the animals. ${\left({\dot{Q}}_{\text{in}}\right)}_{\text{max}}$ corresponds to the capacity required to compensate heat loss—provided that enough instantaneous power is available—for a maximum temperature gap of up to 35°C between inside and outside air temperature. The heating power is set at maximum ${\left({\dot{Q}}_{\text{in}}\right)}_{\text{max}}$ when heating is needed unless the instantaneous power is insufficient.

#### Ventilation

Ventilation rates mainly depend on the age of animals and seasons. Air renewal is crucial along the production cycle for bringing in oxygen and pumping out humidity and potentially harmful gases for animals and farmers, as well as for regulating temperature. During the first three weeks of the production cycle, ventilation rates (${\dot{d}}_{m}$) range from 0.8 and 5 m^3^/h per kg animal [13]. Afterwards, the sensible heat flow strongly increases and ventilation rates rise between 3 and 5 m^3^/h/kg [13]. Power demand for ventilation per unit area of the farm is

$$\begin{array}{c} {\dot{D}}_{V}=m_{a}\;\rho \;{\dot{d}}_{m}\;\Delta P \end{array}$$ (3)

with ${\dot{d}}_{m}$ the ventilation rate per kg animal and Δ*P* the fans’ pressure drop (*i.e*. 900Pa for industrial propeller fans). The ventilation demand per unit surface (${\dot{d}}_{V}$, W/m^2^) is thus comprised in the interval 0.6 − 3.8, which is in line with technical recommendations [12].

#### Thermal regulation

The inside target air temperature *T*_T_ corresponds to optimal growth conditions of chicks and is allowed fluctuating in the vicinity of *T*_T_ in the range *T*_T_±*δT*. Temperature control in the modeling system follows the following rules.

For *T*_in_ < *T*_T_ − *δT*, heating switches on at maximum power, with ${\dot{Q}}_{\text{in}}={\left({\dot{Q}}_{\text{in}}\right)}_{\text{max}}$ until *T*_in_ = *T*_T_ + *δT*. *T*_in_ is then expected to relax due to heat loss (${\dot{Q}}_{\text{cool}}$). While heating the farm, minimum ventilation rates are privileged to reduce heat loss due to temperature gap with the outside.

For *T*_in_ > *T*_T_ + *δT*, ventilation is set at maximum to pump out heat by advection. In case the outside air temperature exceeds *T*_T_ there is no technical way to reach *T*_T_ even with maximum ventilation.

### Power balance of the system

#### Weather report

**Fig 2:**
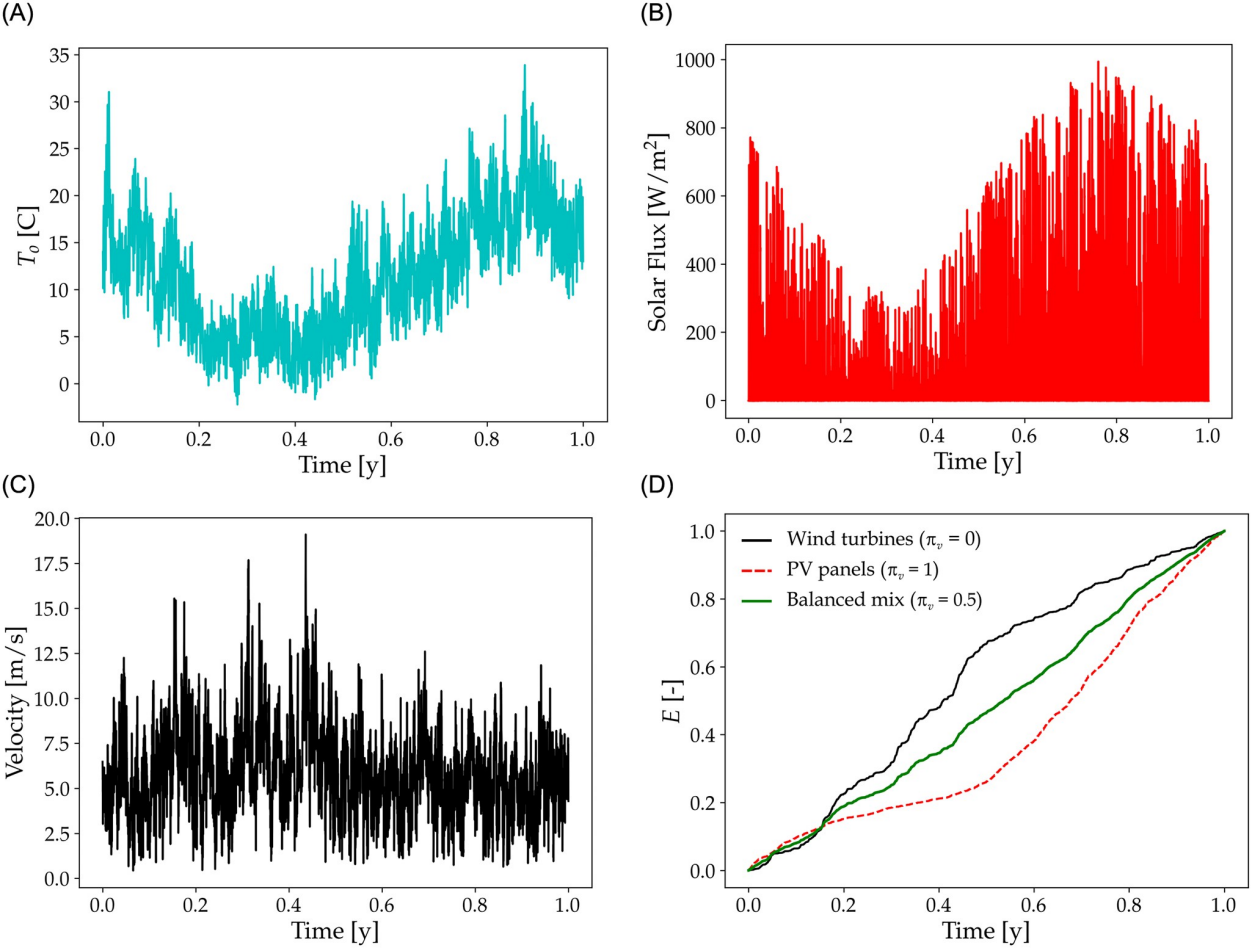
Weather report. Data were recorded in Saclay (France) from September 1^st^ 2013 to August 31^st^ 2014. From left to right and top to bottom: Outside air temperature *T*_o_, solar flux measured at 2 m above ground level and wind speed at 80 m high. The bottom right figure shows the annual energy production profile considering only wind turbines (solid black line), only PV panels (dashed red line) and a balanced mix between wind and PV (*π*_*v*_ = 0.5, solid green line). Energy production is normalized with *E*_ref_ so that annual production equals 1.

Exogenous model parameters are outside air temperature, wind speed and incident solar radiation for renewable electricity generation. Site-specific meteorological and radiative data are provided by the Site Instrumenté de Recherche par Télédétection Atmosphérique (SIRTA) [26] located in the municipality of Palaiseau (2.208 degrees East, 48.713 degrees North) in Paris’ suburban area, about 20 km south of the capital in Ile-de-France region. Such a suburban area might not be the ideal location for setting up a poultry farm, but there is no reason to believe the meteorological and radiative properties that we considered would be statistically much different in slightly more distant rural conditions. Data are considered over a full year, running from September 1^st^ 2013 to August 31^st^ 2014, and report outside air temperature at 2 m above ground level, downwelling solar radiation retrieved by ground-based sun-photometer and wind speed at 80 m derived from Wind Lidar measurements. 80 m is a typical height of modern wind turbines. The time resolution of weather data is one hour, but data were linearly interpolated to one minute, which is the time step of the enthalpic balance simulation. All data were extracted from the SIRTA-Reobs dataset [27]. Mean values of the weather parameters are shown in Table 3, and the complete data series in Fig 2.

**Table 3:** Mean values and standard deviation (*σ*) of key weather parameters. Air temperature (*T*_o_) is measured at 2 m high, wind speed (*v*) at 80 m and solar radiation (*ψ*) at ground level. Data are for the year Sept. 1^st^ 2013 to Aug. 31^st^ 2014, 2.208 degrees East, 48.713 degrees North near Paris, France.

Mean value		Standard deviation	
〈*T*_o_〉 [°C]	11.5 [−2.2;33.9]	*σ*_*T*_ [°C]	6.2
〈*v*〉 [m/s]	5.8	*σ*_*v*_ [m/s]	2.4
〈*ψ*〉 [W/m²]	130	*σ*_*ψ*_ [W/m²]	210

Following the above descriptions of the farm parameters and given the outside temperature we derive the reference annual energy consumption per unit farm area as $E_{\text{ref}}=\sum_{1y}{\dot{D}}_{\text{tot}}\;\delta t$, where ${\dot{D}}_{\text{tot}}$ is total instantaneous power demand of the farm (see Eq 5), and *δt* is the iteration time step. Given the model parameters and data, *E*_ref_ is 85 kWh/m^2^, which is in line with energy consumption reported in [13]. *E*_ref_ is approximately one third of total energy delivered through the animal sensible heat flux, in other words, total annual heating requirement is supplied for 75% by the constant enthalpic heat flux of chicks and for 25% by electricity.

#### Wind power generation

Instantaneous wind power generation from a single wind turbine (*p*_*W*_) is calculated from $p_{W}=\frac{1}{2}\;a\;\rho_{air}\;A\;v^{3}$, where *ρ*_*air*_ is the air density, *v* the wind velocity, *A* the rotor area and *a* the wind turbine energy conversion efficiency, or yield factor. The yield factor *a* varies with wind speed, and is derived from literature data based on semi-empirical models at 80 m height [28]. The yield factor is constrained by minimum and maximum wind speeds of respectively 3 and 25 m/s and is maximizes for speeds between 8 and 10 m/s. Wind turbines are obstacles to air flow and generate air turbulence which reduces energy conversion efficiency. The density of wind turbines (number of turbines per unit land) is constrained by air turbulence. The distancing between wind turbines usually falls in the range between 3 to 10 rotor diameters [29]. We choose the intermediate value of 7*d* (with *d* the rotor diameter) in the direction of prevailing winds and 4*d* in the perpendicular direction. Accordingly, the relationship between the area swept by the wind turbine and the necessary ground surface is *k* = *π*/112.

#### Solar power generation

Solar power from photovoltaic panels depends on solar flux intensity, effective area and energy conversion efficiency of the panels. The effective area of the panels depends on the solar hit angle, which depends on the position of the sun, the orientation of the panel and the horizontal tilt. The chosen angle value is 44° from the horizontal, corresponding to a rough average between the optimal angles for summer and winter. Energy conversion efficiency of panels is function of air temperature, solar flux, air mass (accounting for the thickness of the atmosphere crossed by the incident sunlight) and the type of panels, and is derived from [30]. Here, we consider monocrystalline-silicon modules (*BP 585F mono-Si*) with optimal efficiency of 15%. The real efficiency of the panels is calculated over time from weather input data including solar flux and air temperature and air mass derived from a semi-empirical formula [30] in function of time for the considered geographical position (Saclay, France). For the sake of simplicity, we do not consider reduction of efficiency due to soiling of the panels.

#### Sizing of the renewable power plant

From weather data, we model power production from photovoltaics (*P*_PV_) and wind turbines (*P*_W_). For each power source, we define *E*_*i*_, with *i* standing respectively for PV and W, as the energy produced per year and per unit area: *E*_*i*_ = ∑_1*y*_*P*_*i*_*δt* = ∑_1*y*_*B*_*i*_*p*_*i*_*δt*, with *B*_*i*_ a dimensionless sizing parameter that expresses the surface of renewable power plant required per unit farm area, so that *E*_*i*_ corresponds to total annual energy consumption *E*_ref_. With this definition of *B*_PV_ and *B*_*W*_, each renewable unit is sized to supply the farm’s annual energy demand.

From there, we can compute *B*_W_ = 4.78 and *B*_PV_ = 0.52 which are the necessary ground area occupied respectively by wind turbines and solar panels per unit area of the poultry building to produce *E*_ref_. It is then straightforward to determine power production per unit area of the farm for each power source as *P*_W_ = *B*_W_*p*_W_ and *P*_PV_ = *B*_PV_*p*_PV_.

Total instantaneous power production *P*_VRE_ (Eq 4) is function of *π*_*v*_ (solar fraction of the energy mix, see Fig 1), *N* (dimensionless factor of the power plant, see Fig 1) and *B*_PV_ and *B*_W_:

$$\begin{array}{cc} P_{\text{VRE}} & =\pi_{v}\;N\;P_{\text{PV}}\;+\left(1-\pi_{v}\right)\;N\;P_{W} \end{array}$$ (4)

Fig 2 shows annual cumulative power production from wind and solar panels considering *π*_*v*_ = 0.5. We can easily discern the seasonality of wind and solar power flux in relation to demand. Wind power is better correlated with demand than solar power because both the wind flow and demand maximize during winter.

#### Energy storage and power from the grid

Renewable electricity production can be either used directly by the farm, exported in the grid or stored. The amount of energy stored is denoted as *E*_RES_, and corresponds to the difference between in-going (*P*_RES_) and outgoing (*P*_*S*_) power over time (see Fig 1), *i.e.* $E_{\text{RES}}\left(t\right)=\sum_{0}^{t}\left(P_{S}-P_{\text{RES}}\right)\;\delta t$. Storage is initially empty *E*_RES_(0) = 0. The conversion of stored energy to power is assumed to be instantaneous and entails zero loss. Storage is assumed to be infinite (no upper threshold to storage capacity), and is used as soon as power production is below demand ($P_{VRE}<{\dot{D}}_{\text{tot}}$, see Eq 5). *E*_RES_ = 0 means the storage is empty and consumption cannot exceed instantaneous power production *P*_VRE_.

The poultry facility is also potentially connected to the electricity grid depending on the scenarios (see section *Scenarios*). Power from the grid is *P*_grid_, cumulative power over a given time span is *E*_grid_. *P*_grid_ and *P*_VRE_ are complementary fractions of total available power, and depending on the scenarios, *E*_grid_ is a fraction of *E*_ref_.

### Energy supply impact on the poultry farm operation

The operation of the poultry farm depends on whether the inside air temperature *T*_in_ is within the acceptable range *T*_T_ + *δT*. The relative time over a year that *T*_in_ is within this acceptable range defines the operating time ratio (*τ*) of the poultry building. Using this definition, *τ* reflects the effective productivity of the farm system.

#### Calculation of the inside air temperature (*T*_in_)

Inside air temperature (*T*_in_) at time *t* + *δt* is obtained from *T*_in_ at time *t*. The process to determine *T*_in_(*t* + *δt*) is the following: (*i*) renewable power production *P*_VRE_ (see Eq 4) and total power demand ${\dot{D}}_{\text{tot}}$ are calculated per unit area at time *t* + *δt*:

$$\begin{array}{cc} {\dot{D}}_{\text{tot}} & ={\dot{Q}}_{\text{in}}+{\dot{D}}_{V} \end{array}$$ (5)

${\dot{Q}}_{\text{in}}$ is calculated from Eq 1, with $\frac{\partial Q_{B}}{\partial t}$ being fixed by *T*_in_ at time *t*. The ventilation power demand ${\dot{D}}_{V}$ is calculated following Eq 3. (*ii*) Total power demand ${\dot{D}}_{\text{tot}}$ is compared to produced power *P*_VRE_.

For $P_{\text{VRE}}\geq {\dot{D}}_{\text{tot}}$, power demand is satisfied, and excess power can be stored (*P*_RES_) or exported (*P*_x_) if storage is not allowed:

$$\begin{array}{c} P_{\text{VRE}}={\dot{D}}_{\text{tot}}+P_{\text{RES}}+P_{\text{x}} \end{array}$$ (6)

For $P_{\text{VRE}}<{\dot{D}}_{\text{tot}}$ storage is used with:

$$\begin{array}{c} P_{\text{VRE}}+P_{\text{S}}={\dot{D}}_{\text{tot}} \end{array}$$ (7)

If instantaneous power is below demand and the storage is empty (*E*_RES_ = 0), ventilation is given priority, and the fraction dedicated to heating is ${\dot{Q}}_{\text{in}}=P_{\text{VRE}}-{\dot{D}}_{\text{V}}$. In this case, the ventilation is set at minimum (see Table 2) to allow minimizing heat loss from air renewal and maximizing heating. (*iii*) The effective temperature *T*_in_ at *t* + *δt* is calculated from Eq 1, where ${\dot{Q}}_{\text{in}}$ is fixed.

Depending on the scenario (see section *Scenarios*) power from the grid can compensate for inadequate *P*_VRE_ to reach the demand ${\dot{D}}_{\text{tot}}$. Eq 7 is then replaced by Eq 8:

$$\begin{array}{c} P_{\text{VRE}}+P_{\text{grid}}={\dot{D}}_{\text{tot}} \end{array}$$ (8)

#### Operating time ratio of the poultry farm

We address the potential impact of energy supply on the farm operation by considering the inside air temperature *T*_in_ and ventilation. To comply with the production conditions, ventilation needs to be at least equal to a minimum air renewal flow rate and *T*_in_ needs to be in the vicinity of target air temperature *T*_T_ + *δT* over a minimum duration *D*.

We define the operating time ratio of the poultry farm (*τ*) as the normalized annual duration over which the production conditions are respected. *τ* = 1 corresponds to continuous operation of the poultry facility, *i.e.* when both target temperature and ventilation are satisfied with no interruption. *τ* = 0 corresponds to zero satisfaction of at least one of the two conditions.

Naturally, *τ* is sensitive to the length of the time window *D*. A short time window implies that the cumulative effects of sub-optimal heating on poultry production are minimal. All things being equal, the longer the time window *D*, the more the satisfaction of the operating conditions is constraining, and thereby, *τ* is low. However, in absence of clearly defined heat stress constraints on poultry growth in the literature, we set the value *D* to 1 hour, which is the time step of our simulation, and the least constraining condition possible. For detailed discussion of the impact of *D* value on *τ*, see section *Supp. Mat./Minimum continuous duration*.

### Scenarios

We study four scenarios which are summarized in Table 4. Scenarios *A*.*i* explore various combinations of power supply from renewable sources and the grid, and differ in term of storage. Storage is authorized in *A*.1 and prohibited in *A*.2. These two scenarios explore the impact of VRE penetration and storage on the operating ratio *τ* without oversizing of the renewable power plant (Eq 9). *E*_grid_ ranges as a fraction of *E*_ref_ from 0 to 1, and *N* is the fraction of *E*_VRE_ to *E*_ref_ so that *E*_tot_ = *E*_ref_.

$$\begin{array}{cc} E_{\text{VRE}}+E_{\text{grid}} & =E_{\text{ref}} \end{array}$$ (9)

**Table 4:** Scenarios summary. In scenarios *A*.*i* annual energy demand (*E*_ref_) is supplied by a mix of VRE and power from the grid. In scenarios *B*.*i* there is no connection to electrical network (*P*_grid_ = 0), and only renewable production is allowed to vary. For both *A*.1 and *B*.1, renewable power production can be stored, while it is prohibited in *A*.2 and *B*.2.

Scenarios	*E*_grid_ + *E*_VRE_ = *E*_ref_	*P*_grid_ = 0
Renewable power production can be stored in *E*_RES_	*A*.1	*B*.1
Surplus power storage is prohibited (*E*_RES_ = 0)	*A*.2	*B*.2

Scenarios *B*.*i* exclude power supply from the grid (*E*_grid_ = 0) and explore the effect of the sizing of the renewable power plant (*N*) on *τ* with and without storage. Storage is authorized in *B*.1 and prohibited in *B*.2. The sizing factor *N* is set to vary from 0 to 2; *N* = 2 meaning that the annual renewable energy production is twice as *E*_ref_.

In scenarios *A*.2 and *B*.2 (no energy storage), instantaneous power is either immediately used or exported (*P*_x_). In scenarios *A*.1 and *B*.1, stored energy is *E*_RES_ and increases when $P_{\text{VRE}}>{\dot{D}}_{\text{tot}}$.

## Results

**Fig 3:**
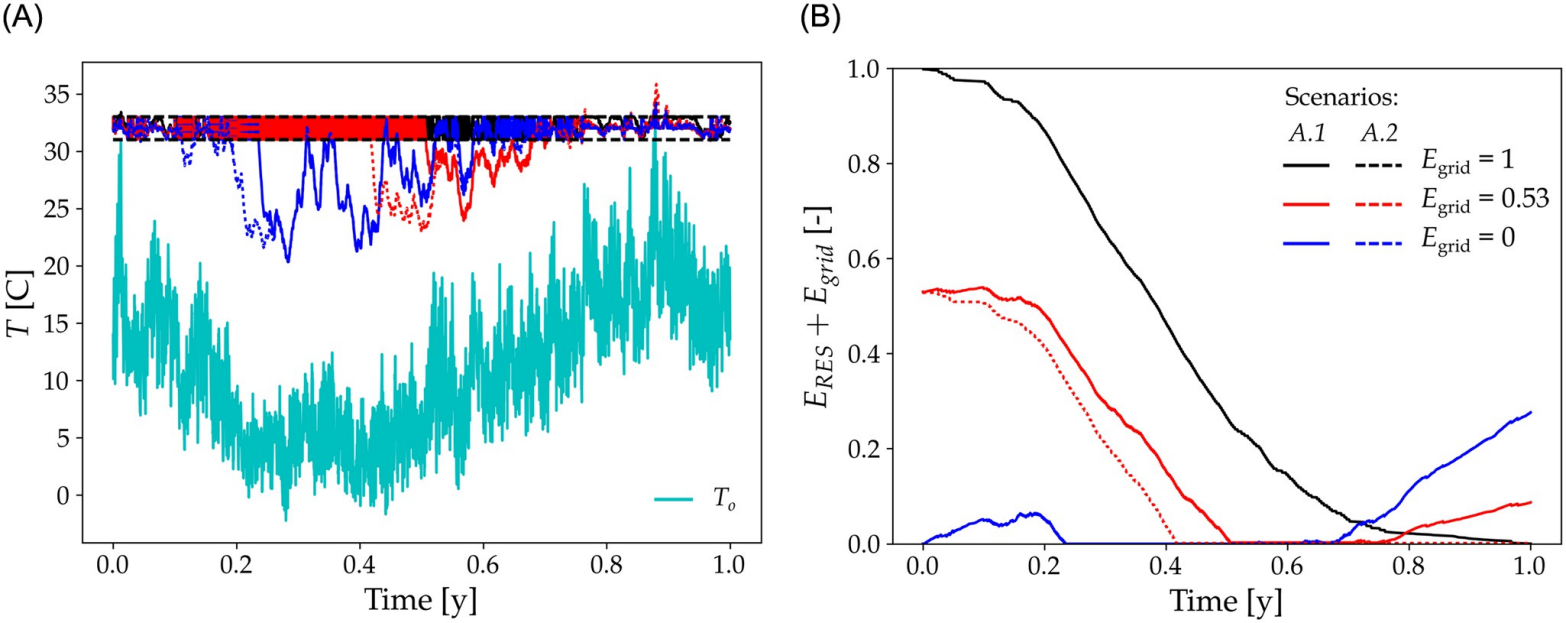
Temperature dynamics and energy storage over time. Left is outside *T*_o_ (cyan) and inside temperatures *T*_in_ in function of time, using scenario *A*.1 (solid lines) with storage facility and scenario *A*.2 (dashed lines) without storage facility. *E*_grid_ = 1 in black, 0.53 in red and 0 in blue. Respective *τ* are 1, 0.84 and 0.74 for scenario *A*.1, and 1, 0.74 and 0.67 for scenario *A*.2. In all cases, solar fraction in the energy mix is *π*_*v*_ = 0.5 and total input energy is equal to *E*_ref_. The amount of VRE is then given by *E*_VRE_ = *E*_ref_ − *E*_grid_. Horizontal black dashed lines show the temperature target interval. Right are the corresponding instantaneous available energy stock, from VRE and from the grid *E*_*S*_ = *E*_grid_ + *E*_RES_ in function of time using the same color code.

We show in Fig 3 the evolution in time of the outside and inside air temperature (left panel) in relation to the energy supply mode of the farm (right panel) in scenarios *A*.1 and *A*.2, *i.e.* when total energy*E*_tot_ = *E*_ref_ = *E*_VRE_ + *E*_grid_.

The black line in the right panel shows the profile of unconstrained annual energy demand, *i.e* full satisfaction of the energy demand (*E*_grid_ = 1, meaning *N* = 0). In this case, *T*_in_ = *T*_T_±*δT* throughout the year in both A.1 and A.2 scenarios—respectively solid and dashed black lines—and *τ* = 1. Decreasing *E*_grid_ (red and blue lines) implies increasing shares of VRE in total supply (*N* > 0). This causes deviation in *T*_in_ from *T*_in_ = *T*_T_±*δT* especially during winter (lowest outside air temperatures). With *E*_grid_ = 0 (blue line, corresponding to *N* = 1) and although, by definition, the renewable energy production equals the annual demand, *T*_in_ is below *T*_in_ = *T*_T_±*δT* for long periods of time. Energy storage always improves the temperature control (solid against dashed lines in Fig 3) but is not a sufficient condition for full satisfying the energy demand throughout the year because power demand and supply are strongly out-of-phase. Indeed, with decreasing shares of energy supply from the grid, the periods of energy deficits in particular during winter increase (i.e. *T*_in_ increasingly diverges from *T*_T_). Correlatively, the amount of unused energy by the end of the year also increases. For instance, in the case *E*_grid_ = 0.53 (VRE supplies 0.47 of the annual energy demand, red line), about 10% of *E*_ref_ is stored but remains unused by the end of the year.

In the case *E*_grid_ = 0, VRE meets the demand only during half the year (over the first 25% and last 30% of the annual time). For the rest of the year, *T*_in_ is far below the target temperature *T*_T_.

**Fig 4:**
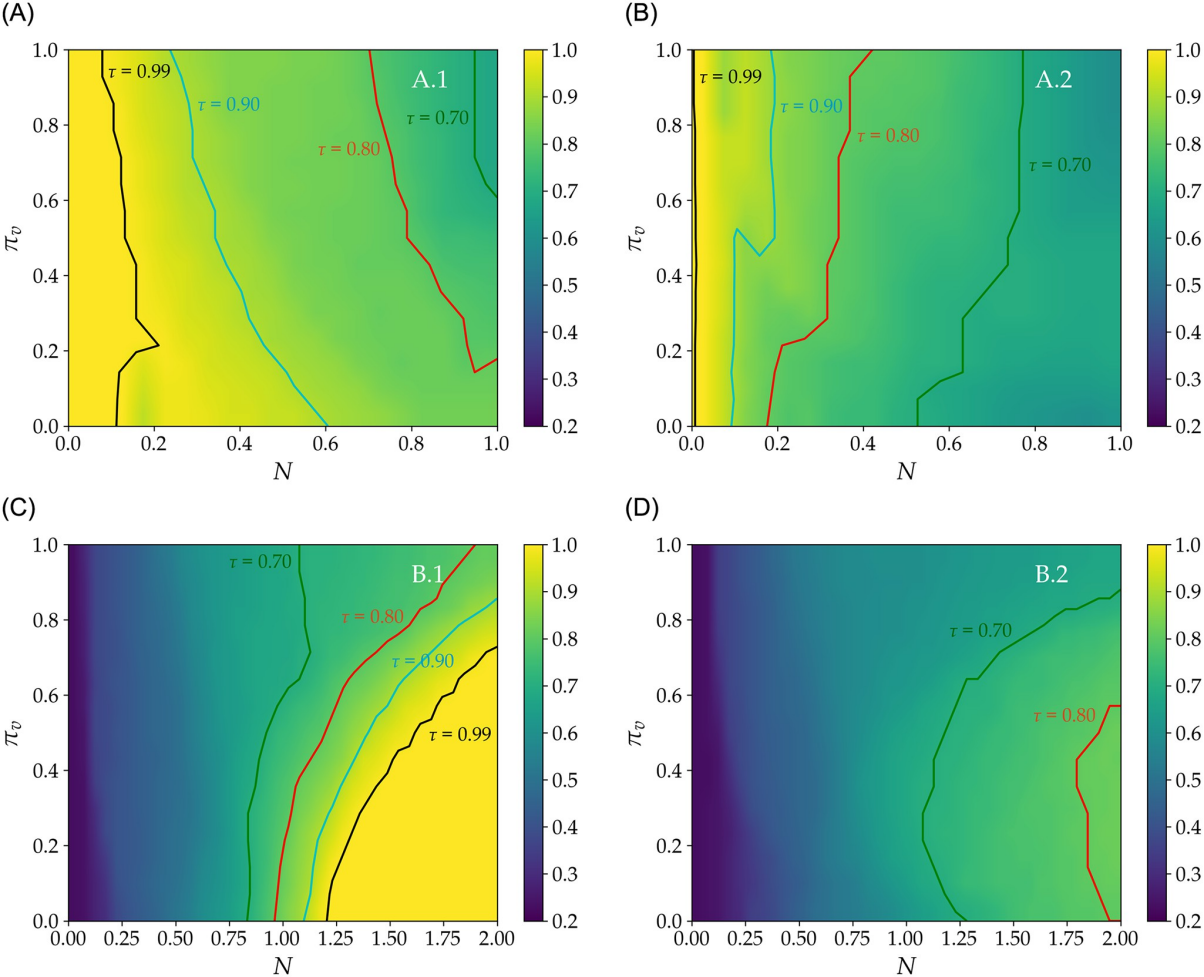
Annual operating time ratio (*τ*) of the poultry building in function of the annual fraction of renewable to reference energy demand (*N*) and energy mix (*π*_*v*_). *π*_*v*_ is the share of solar to solar and wind power generation (*π*_*v*_ = 0 means 100% wind and *π*_*v*_ = 1 means 100% solar). *N* = 1 corresponds to VRE equaling the annual reference energy demand of the farm. In scenarios *A*.1 and *A*.2, total energy is *E*_tot_ = *E*_grid_ + *E*_VRE_ = *E*_ref_. In scenarios *B*.1 and *B*.2, power supply from the grid is null (*E*_grid_ = 0). The green (respectively red, cyan and black) line shows *τ* = 0.70 (respectively 0.80, 0.90 and 0.99).*A*.1 and *B*.1 (respectively *A*.2 and *B*.2) scenarios allow (respectively forbid) renewable energy storage (see Table 4).

Let-us now generalize the derivation of *τ* in various conditions. In Fig 4, we show the variation of *τ* (color grid) in function of the energy mix (*π*_*v*_) and of the fraction of VRE in *E*_ref_ (*N*). Equivalent *N* and *π*_*v*_ combinations are directly indicated in Fig 4 for *τ* equaling 0.7, 0.8, 0.9 and 0.99.

In scenarios *A*.1 and *A*.2 (constant total energy *E*_tot_ = *E*_VRE_ + *E*_grid_ = *E*_ref_), we find that *τ* varies from 1 to below 0.6 depending on the energy mix (*π*_*v*_), VRE sizing (*N*), and the possibility to store energy. In scenario *A*.1 (possible energy storage), VRE penetration for a given *τ* increases when *π*_*v*_ decreases. This is because of the favourable timing between wind power generation and power demand during autumn and winter (Fig 2). Indeed, wind power generation maximizes slightly before demand, allowing for relatively high direct power penetration and storage of excess power for subsequent use. Inversely, solar power production is rather produced during summer, when the energy needs of the farm are minimal. Accordingly, when *π*_*v*_ increases, production and demand get increasingly decoupled in time, leading to energy shortages during winter and unused energy surplus during spring and summer. Inversely, in Fig 4-*A*.2 (without storage), increasing *π*_*v*_ appears to favor higher direct penetration of VRE for a given *τ*. This is because the good match between solar power generation and power demand for ventilation during spring compensates for the mismatch during winter. In absence of storage, wind power generation loses its relative advantage compared to solar power, and *τ* dramatically decrease when *N* = 1 to below 0.6 regardless of the energy mix.

In Fig 4-*B*.1 and 4-*B*.2, the size of the power plant varies with *N*, and there is no power supply from the grid. In Fig 4-*B*.1 (possible energy storage), achieving *τ* > 0.7 requires oversizing the renewable power plant, and *τ* ≈ 1 is possible for *N* > 1.25, and *π*_*v*_ = 0. In general, for *τ* > 0.8, Fig 4-*B*.1 suggests that increasing *π*_*v*_ induces oversizing of the renewable power plant (*N* > 1). Indeed, for *π*_*v*_ = 0 and *N* ≳ 1.25, *τ* ∼ 1, whereas *τ* ∼ 0.85 even for *N* = 2 when *π*_*v*_ exceeds ≈0.7. In Fig 4-*B*.2 (no storage), *τ* is sensibly lower than in *B*.1 and maximizes at ≈0.8 when the renewable power plant is oversized twofold—with *N* = 2, *i.e.* *E*_VRE_ = 2*E*_ref_) and *π*_*v*_ = 0.5.

**Fig 5:**
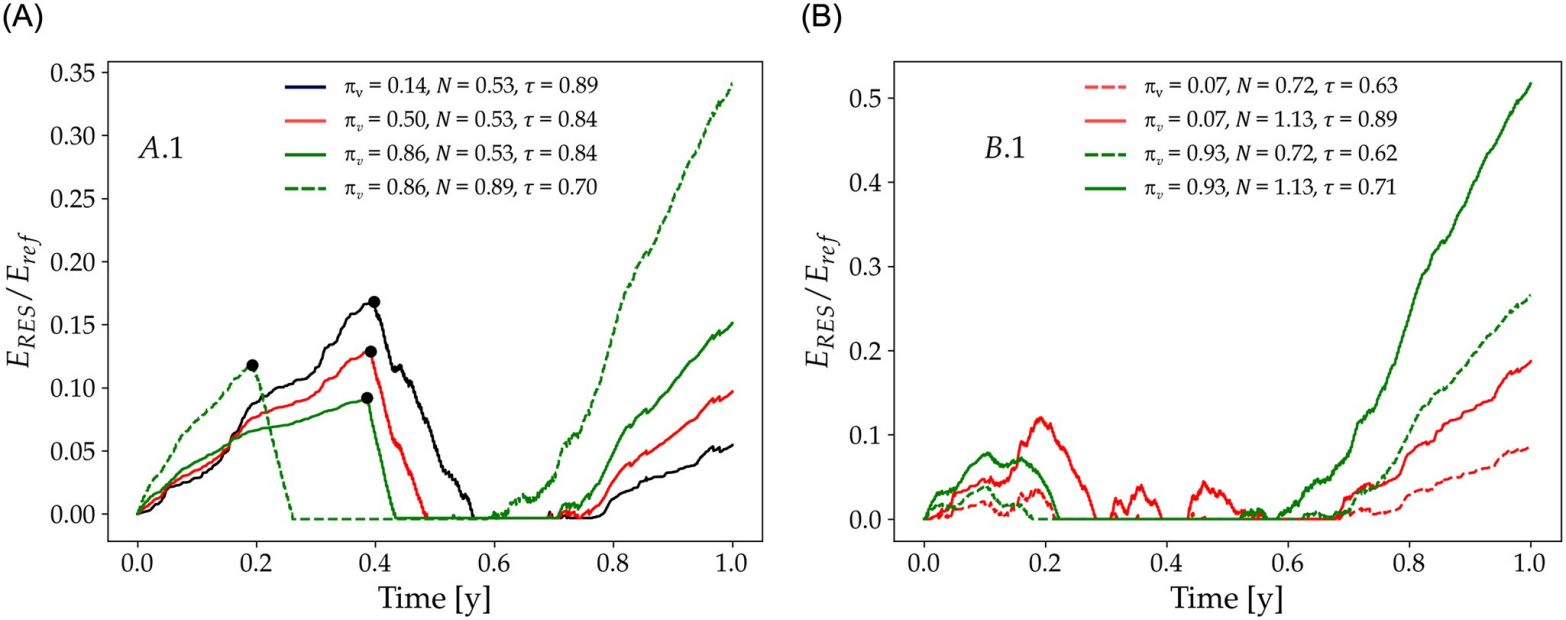
Normalized stored energy *E*_RES_ for scenarios *A*.1 (left) and *B*.1 (right) in function of time. Scenario *A*.1 highlights the value pairs (*π*_*v*_, *N*) = (0.14, 0.53) in black which leads to *τ* = 0.89; with (0.50, 0.53) in red *τ* = 0.84); with (0.86, 0.53) in green *τ* = 0.84; and with (0.86, 0.89) in dashed green *τ* = 0.70. Black dots indicate the moment *E*_grid_ reaches the authorized threshold. Scenario *B*.1 highlights the value pairs (*π*_*v*_, *N*) = (0.07, 0.72) in dashed red line which leads to *τ* = 0.63, with (0.07, 1.13) in solid red line *τ* = 0.89, with (*π*_*v*_, *N*) = (0.93, 0.72) in dashed green line *τ* = 0.62, and with (0.93, 1.13) in solid green line *τ* = 0.71.

In order to assess the minimum energy storage capacity required in scenarios *A*.1 and *B*.1, we consider the stored energy *E*_RES_ as a fraction of *E*_ref_ over time, see Fig 5. Note that at each time step, *E*_RES_ is the available storage calculated as the cumulative difference over time between instantaneous power generation and demand. In scenarios A.2 and B.2, *E*_RES_ is null by definition.

Let-us first focus on scenario *A*.1 (see Fig 5-A.1) which combines instantaneous power use, renewable energy storage and freely available electricity from the grid (*E*_grid_). At each time step, if the instantaneous power generation is below demand, it is complemented by power supply from the grid (*E*_grid_) until this latter reaches the threshold defined by *N* (*i.e.* 1 − *N* is the allowable annual share of electricity from the grid to total demand). If instantaneous power generation exceeds demand, power from the grid is zero and excess renewable power is stored as *E*_RES_. The moment the threshold of *E*_grid_ is reached is indicated by the black dots in Fig 5-*A*.1. After reaching this threshold, supplement power is supplied by *E*_RES_ until exhaustion of this latter.

Fig 5-*A*.1 shows that when power production is supplied for one half by the renewable power plant (*N* = 0.53) and is mostly driven by wind (*π*_*v*_ = 0.14, black line), *E*_RES_ allows achieving *τ* of 0.89 while unused storage by the end of the year is relatively small, about 5%. With solar-driven power generation (*π*_*v*_ = 0.86, green line), *E*_RES_ is early exhausted leading to a long-term (seasonal) energy deficit that increases with *N* (solid vs dashed lines in Fig 5-*A*.1). The unused storage at the end of the year increases with *π*_*v*_ and *N*, implying that direct penetration of VRE can increase without decrease in *τ* by adapting the energy mix. Note that for *N* = 0.89, the unused energy stored at the end of the year is about threefold higher than in the case *E*_grid_ = 11%.

In scenario *B*.1, power supply from the grid is prohibited and total energy production increases with *N*, while excess renewable power can be stored (${\dot{E}}_{\text{RES}}>0$). Fig 5-*B*.1 shows that both *τ* and unused *E*_RES_ increase with *N* (solid against dashed lines) and greatly vary with the energy mix. Indeed, wind-driven power generation (low *π*_*v*_, red lines) leads to higher *τ* and lower unused *E*_RES_ by the end of the year compared to solar-driven power generation (high *π*_*v*_, green lines).

We observe that the relationship between *N*, *τ*, *π*_*v*_ and unused *E*_RES_ is not linear. For instance, for *N* = 0.72 (dashed red and green lines), wind and solar-driven power production lead to equivalent *τ* but to more than a twofold difference in the amount of unused energy by the end of the year. This indicates that the amount of energy used in heating the building without reaching the target temperature is higher for wind-driven energy mix (low *π*_*v*_). However, by increasing the size of the power plant by approximately 50% (*N* increase from 0.72 to 1.13), *τ* increases substantially under wind-driven power generation (from 0.63 to 0.89) but only moderately (from 0.62 to 0.71) under solar-driven power generation. In parallel, the unused storage by the end of the year doubles in both cases but with a contrasting profile over the winter period. In the case of solar-driven power generation, *E*_RES_ = 0 between time 0.3 and 0.7 of the simulation —which is a seasonal effect—whereas for wind-driven production *E*_RES_ shows a typical saw-tooth profile (Fig 5-*B*.1). This is due to the more favorable timing between wind compared to solar power generation and demand, and highlights that oversizing the power plant is only relevant when power generation occurs prior to use.

## Discussion

The transition from fossil energy (stocks) to renewable power (flows) is today a major sustainability challenge and reintroduces an old (pre-industrial) constraint in the use of resources. This constraint is the fixed power intensity of renewable flows, *i.e.* which is determined by external conditions, in contrast to the power extraction intensity from stocks, which can vary arbitrarily. The shift from stocks to flows [31] may threaten the functioning of a demand system due to constrained short-term power availability.

Wind and solar flows are characterized by strong random variability at hour scales and periodical occurrence at daily and seasonal scales. This complex variability introduces multiple constraints in the energy supply dynamics of systems [32], including stability challenges for renewable power absorption in electric grids [33], and contrasts with the objective of continuous production functions [34, 35], which among other systems, is the case of industrial agriculture.

We have introduced the quantity *τ* defined as the fraction over a year that a system (here a poultry farm) can operate under VRE supply. This quantity consists in evaluating the effect of power supply fluctuations on the system’s operating conditions and, thereby, the aggregate duration of potential disruptions. However, the timing of the system operation may vary depending on the power source. In general, pure solar (*π*_*v*_ = 1) and pure wind (*π*_*v*_ = 0) power supply systems display contrasted behaviours in terms of the timing they satisfy the demand. Under pure solar power supply, *T*_in_ allows for quasi-continuous operation of the poultry system during spring and summer. Under pure wind power supply, *T*_in_ allows for discontinuous system operation (roughly per one-week period) but throughout the year. The system can better adapt to fluctuations by modulating the degrees of freedom that represent the energy mix and storage capacity. For instance, mixed power supply (*π*_*v*_ = 0.5), can allow increasing *τ* as well as improving the continuity of the system’s operation compared to pure solar or wind systems. The improvement is all the more significant when seasonal energy storage is considered.

**Fig 6:**
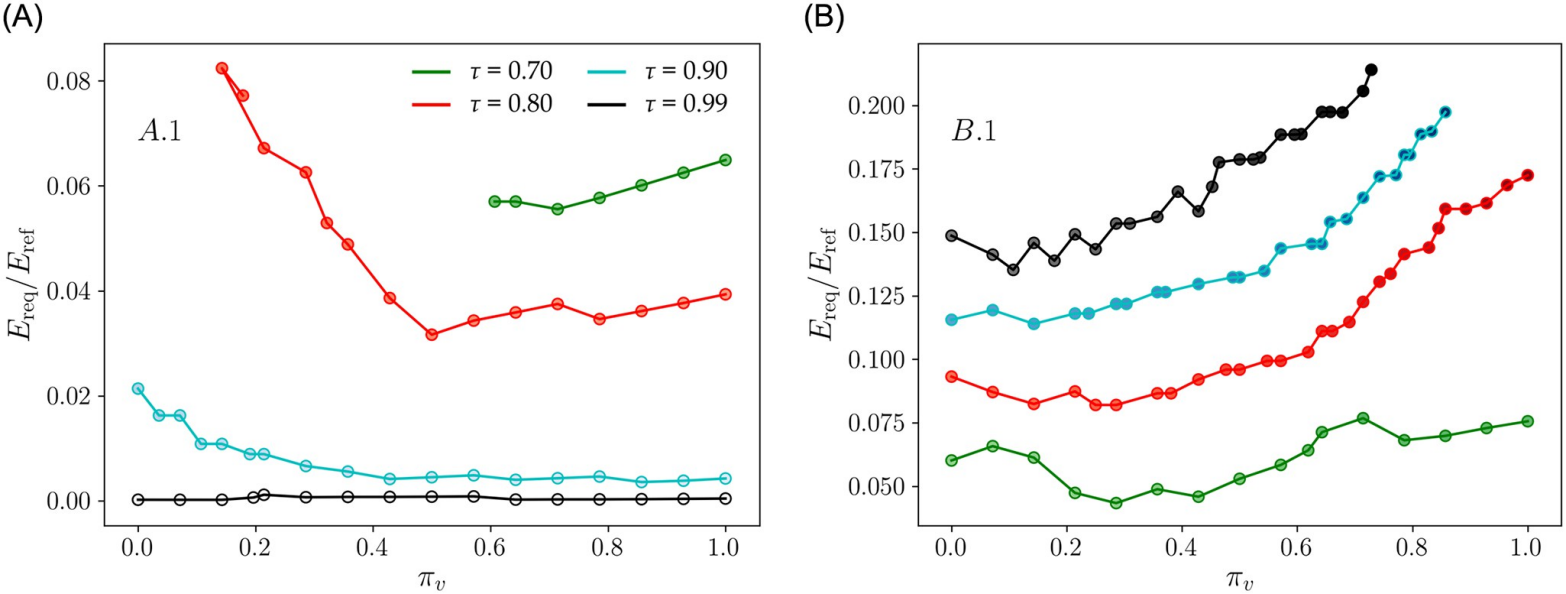
Minimal energy storage capacity (*E*_req_) required to maintain constant *τ* in function of the energy mix in scenarios *A*.1 and *B*.1. *τ* = 0.7, 0.8, 0.9 and 0.99, for respectively green, red, cyan and black lines, and using the same color code as in Fig 4-*A*.1 and 4-*B*.1. Note that *N*-values are not conserved along each curve but can be directly extracted from Fig 5-A.1 and 5-B.1.

Indeed, storage allows using asynchronous power generation which is synonymous to providing the demand system with a fixed boundary condition. From an operational standpoint, it can be interesting to set a target *τ* and size the storage capacity accordingly. To this end, the required storage capacity (*E*_req_) can be defined as the minimum necessary energy storage for maintaining *τ* at a constant value. Accordingly, *E*_req_ is the fraction of *E*_RES_ that excludes unused storage capacity. As shown in Fig 5, *E*_RES_ can reach its maximum value at the end of the year depending on the value pair (*π*_*v*_, *N*), but much of this storage corresponds to unused energy, and therefore, to unused storage capacity. To better discuss this issue, Fig 6 shows *E*_req_ in function of *π*_*v*_ in scenarios *A*.1 and *B*.1 for the four values of *τ* indicated in Fig 4, and highlights that *E*_req_ is a rather small fraction of *E*_ref_. For instance, the black line in Fig 6-A.1 indicates that with minimal storage (less than 0.1% of the annual energy requirement), it is possible to integrate a non-negligible quantity (above 10%, see Fig 4-*A*.1) of VRE in annual supply, while maintaining a quasi-optimal *τ* (*i.e.* *τ* = 0.99). Besides being a function of the energy mix (*π*_*v*_), *E*_req_ is in all cases a relatively small fraction of *E*_ref_. The red line in Fig 6-A.1 indicates that storage capacity between 4 and 8% of *E*_ref_ allows achieving *τ* of 0.8 even with respectively 75% to 100% VRE penetration. In contrast, if storage is prohibited, high penetration of renewable power—*e.g.* above 60%—involves a drastic decrease in *τ*—below 0.7—regardless of the energy mix, see Fig 4-*A*.2.

We also highlight that when power supply from the grid is prohibited (scenario *B*.1) but storage is allowed, *E*_req_ increases with *τ*, and—in most cases—with *π*_*v*_ up to a threshold of about 20% of *E*_ref_ (see Fig 6-*B*.1). However, we can observe that an increase in the share of solar power (increase in *π*_*v*_) drives up both the storage requirement and the sizing (*N*) of the VRE plant. For example, reaching *τ* = 0.99 when *π*_*v*_ = 0.71 (see black line in Fig 6-B.1) requires on the one hand doubling the renewable power plant (*N* = 2) and, on the other hand, storing 25% of *E*_ref_ against only a moderate oversizing (*N* = 1.25) and storage of roughly 14% when *π*_*v*_ is about 10%. This example highlights the implications of the VRE mix on all dimensions of the power production system, as well as the fact that setting a target *tau* may be highly relevant under intermittent power supply.

In sum, our results provide an overview of feasible combinations and trade-offs between key energy system components and allow evaluating the implications of different power supply configurations, which is barely done at local scales in the scientific literature. It is interesting to note that under the most favourable energy mix combinations and with a storage capacity of only 0.04% of *E*_ref_, it is possible to introduce more than 10% of VRE in the energy mix without any loss in *τ*. The share of VRE in total power supply can even increase at 60% with storage of 0.4% if we agree to decrease *τ* at 90%.

To highlight the insights of the study at a higher level of generalization, it is interesting to note that estimated storage requirements in energy systems at scales overwhelmingly larger than the single poultry farm in our study—such as the scale of power supply to the European continent [20]—are fairly comparable with our results. For instance, the estimated storage requirement for 100% VRE supply in the case of Europe is reported to vary between 0.15% and 20% of the annual demand, which is also the case in our simulations (Fig 5-*B*.1, black line). Similarly, in line with our results, the storage requirement estimated at such large scales also increases with the share of solar power in VRE [20]. The relationship between power plant oversizing and storage requirements is also fairly comparable across scales. Indeed, at both scales, satisfying the demand with reduced storage requires oversizing the power production plant. In the case of whole Europe, a power plant oversizing by 50% allows lowering the long-term storage requirement at only 1% of the annual demand [36], which is close to the result obtained in our study.

Note that the starting date of the simulations influences the profile of power demand compared to production and, thereby, of the storage requirement to achieve a given *τ*. The configuration that minimizes storage for a given *τ* is when excess power generation and deficits take turns, thus resulting in an intensive turnover of a relatively small storage capacity over short and successive periods of time. This is the case in the presented results, *i.e.* simulations starting in late summer (*e.g.* September 1st). Indeed, in late summer, the demand is a relatively small fraction of instantaneous power production due to mild ambient temperatures and relatively high renewable energy intensity. As shown in Fig 8 in Section *Supp. Mat/Starting date…* in S1 File, the storage requirement is higher when the simulation starts on January 1st (left panel) and April 1st (right panel) instead of September 1st. Of course, the results also depend on the meteorological input data which define both the energy demand and the renewable energy available for production. As these data are recorded in northern suburban France, our results should be regarded as representative of a poultry farm installation in regions that globally share the same average meteorological conditions, *i.e.* mid-latitude countries located relatively close to the ocean.

Regardless of the scale of the analysis, storage is a major limiting factor in the energy transition both in terms of duration and total capacity. This is due to the low energy density of most storing technologies and to energy dissipation during storage cycles, which both drive up primary energy requirements per unit final energy use [37]. The most promising type of large-scale storage capacity is probably hydrogen systems via the classic chain of “power to gas to power” using water electrolysis and a fuel cell [38]. Per unit mass, the energy density of hydrogen is about 100 MJ/kg, *i.e.* threefold higher than typical gasoline [39], and hydrogen (associated with oxygen in the form of water) is among the most abundant elements on Earth. However, two persisting technical issues are the constraining conditions of low temperature and high pressure required to reduce the volume-to-mass ratio of hydrogen storage, and the low energy efficiency in hydrogen conversion chains which is today about 30–40% [39]. The lower the conversion efficiency, the higher the energy dissipation and, therefore, the amount of primary energy required per unit storage. There is currently substantial research focus on technical storage improvements, but technical aspects of storage are out of scope in our analysis. For the sake of simplicity, we have considered zero loss in storage, *i.e.* 100% conversion efficiency, to eliminate an unnecessary model variable and emphasize the relationship between *π*_*v*_, storage requirement and *N*. From an energy transition perspective, gains in energy efficiency of storage are highly beneficial as they allow reducing the renewable power capacity necessary to install. The option of reducing final energy demand is equivalent to, and technically less challenging than, increasing efficiency and, in the case of livestock buildings, it can rely on relatively simple optimization systems such as heat loss abatement during ventilation based on heat exchange systems [40]. Storage requirements can also be potentially reduced by taking into account network connections among locations with different solar radiation and wind speed profiles as well as multiple energy demand profiles, which can both potentially affect the composition and improve the stability and direct VRE penetration.

Among the novel theoretical insights of our study, we stress the trade-off between the type of energy supply to a demand system and its operating efficiency. In general, industrial livestock farms greatly rely on controllable power flows for heating and ventilation in order to optimize animal productivity [41]. Consequently, power supply is an indispensable input to metabolic efficiency gains in livestock, in particular in relation to feed conversion [42]. Power supply from current sources results from historical adaptation given environmental constraints including fixed versus variable boundary conditions [31]. In current industrial systems, production functions are well adapted to a specific energy regime, and this adaptation leads to specialization as a strategy for optimizing resource use through economies of scale [43]. However, the consequence of high adaptation is lack of adaptability [44], meaning that a change in boundary conditions can entail critical degradation or even a collapse of the system’s technical performance. In the case of the poultry sector, adaptation has led to vertical specialisation of production stages involving the physical separation of birds in specialized units according to age and size. Chicks have very high temperature requirements due to their high body surface-to-volume ratios, and these requirements are hard to meet in absence of a reliable energy supply. As long as chicks are grown together with bigger poultry, the required heat flux is guaranteed by the bigger birds through contact and brooding given that the enthalpic flux increases with weight [45]. In contrast, when chicks are grown separately, the reliability in heat supply relies on fixed boundary conditions and is lost when switching to flow boundary conditions [31]. Such a switch equates to losing efficiency in the production engine and leads to the following dilemma. Either regaining adaptability by reducing the system’s specialization degree, or exploring adaptation strategies by optimizing the available degrees of freedom of the demand system. The first option is a long-term process that potentially requires a full system redesign [46], and probably a complex simulation of economic and societal cascade effects. In this work, we have explored the second option by assessing the conditions of adaptation of the system to new boundary conditions. This includes assessing the dependence of energy demand on controllable flows and the impact of on-site VRE mix on the system’s operating patterns and storage requirements. Moreover, the impact of climate change on local meteorological conditions would be relevant to account for in more complex prospective scenarios. Nonetheless, it is important to note that accounting for climate change by simply increasing the temperature in the simulations would be convenient but not representative of the actual meteorological conditions that are expected. Quantifying the impact of climate change on the results presented here is the object of future work, and should necessarily take into account projected change in the conditions of renewable power generation.

## Conclusion

We quantify the impact of VRE supply constraints on an energy demand system, and assess the conditions of adaptation of the system to flow boundary conditions. It is impossible to obtain the same *τ*, i.e. unchanged operating conditions, as with stock boundary conditions without oversizing the renewable power production plant and storing a considerable share of the annual energy demand. Higher penetration of VRE with lower storage requirements and moderate oversizing is possible by reducing *τ*, meaning by introducing a seasonality in the production cycle of specialized systems as it is the case in open-field vegetal production systems in most climates.

## Supporting information

S1 File (PDF)
