# Supplementary material for: Variable renewable energy penetration impact on productivity: A case study of poultry farming
Source: PLoS One. 2023 Oct 2;18(10):e0286242. doi: 10.1371/journal.pone.0286242 (PMC10545111; doi:10.1371/journal.pone.0286242)
Supplement: S1 File — (PDF) [file pone.0286242.s001.pdf]

Supplementary Material

Minimum continuous duration

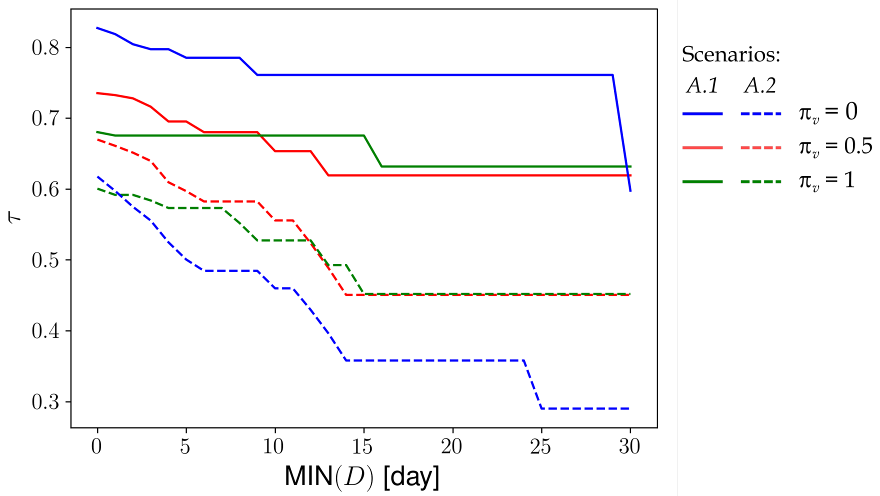

Fig 7. Operating time τ under renewable power supply ($E_{\text{grid}} = 0$, $N = 1$) in function of the duration D with storage (scenario A.1, solid lines) and without storage (scenario A.2, dashed lines). D is the minimum continuous duration for which the target temperature is respected ($T_{\text{in}} = T_{\text{T}} \pm 2°C$). The solar fraction of the energy mix (π_v) is respectively 0% (in blue), 50% (in red) and 100% (in green).

In Fig. 7, we show the operating time ratio τ as a function of the minimum continuous duration (D) over which the target temperature and ventilation conditions of the poultry farm are respected, with and without energy storage (scenario $A.1$ and $A.2$ respectively). The simulation considers 100% VRE ($E_{\text{grid}} = 0$) and energy mix variation from pure wind ($\pi_v = 0$) to pure solar ($\pi_v = 1$) with intermediate $\pi_v = 0.5$. As expected, τ decreases with D, and the decrease is the steepest in absence of storage ($A.2$ dashed-lines) regardless of the energy mix. The decrease in τ also depends on the energy mix (π_v) due to the difference in seasonality between wind and solar power production (on this matter, see further analysis based on Fig. 4). Note that for VRE dominated by wind ($\pi_v = 0$), the decrease in τ is the steepest without storage, and the mildest with storage, suggesting that wind production coupled with storage has the best adaptation capacity to the energy demand. In contrast, without storage, medium to high share of solar power in the energy mix ($\pi_v > 0.5$) appears to be more favorable than pure wind ($\pi_v = 0$). All the results presented hereafter are obtained for $D = 1$ hour, which is the least constraining value for the energy functioning of the farm.

Starting date of the simulation

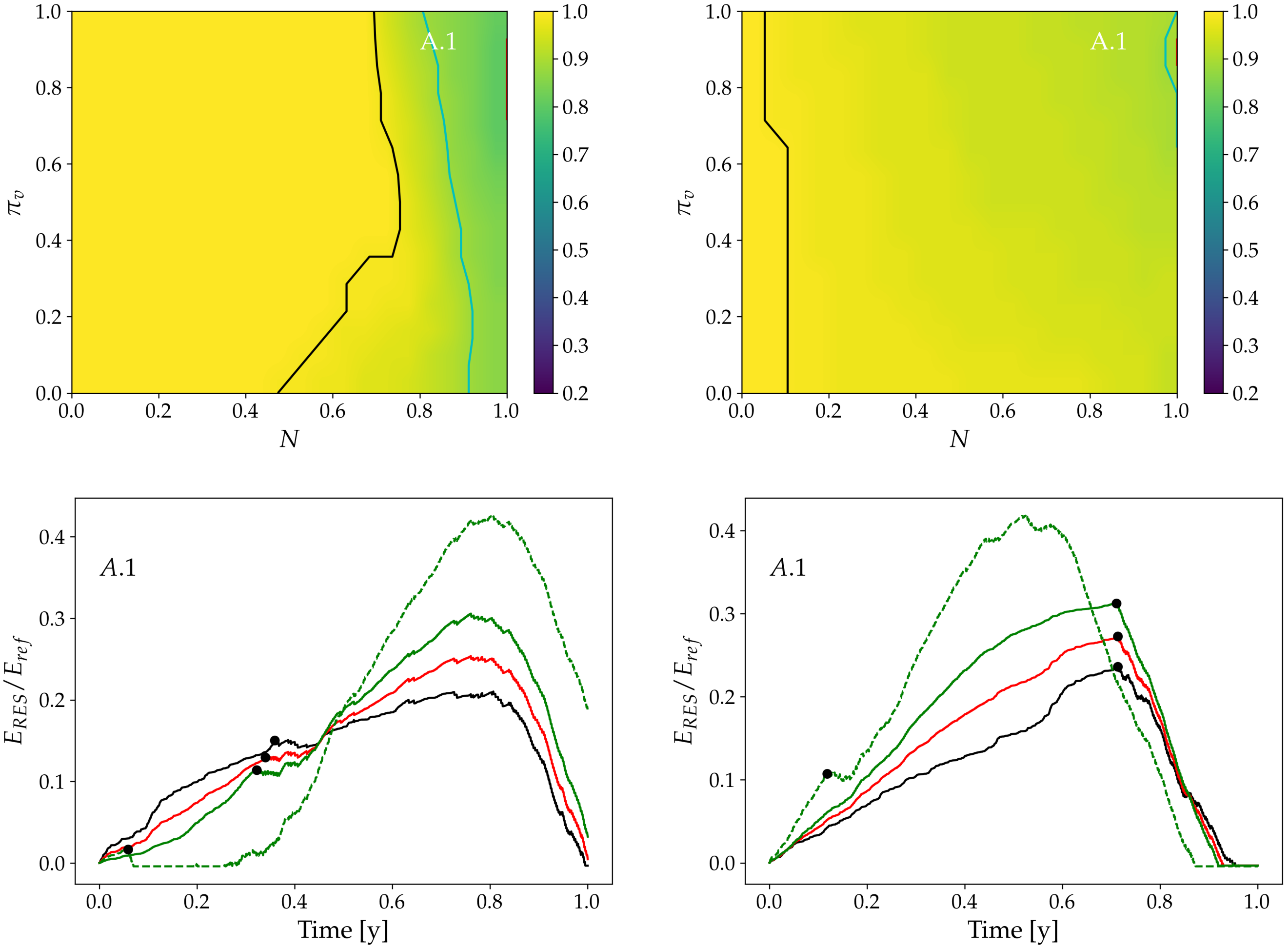

Fig 8. Chick farm annual use rate τ (top) and stored energy (bottom) for scenario $A.1$, with initial time the first of January (left) and the first of April (right). For top figures, same color code as in Fig. 4 with $\tau = 0.99$ (black line) and 0.90 (cyan line). For bottom figures, same color code as Fig. 5, with $(\pi_v, N) = (0.14, 0.53)$ in black which leads to respective $\tau = 1$ (left) and 0.96 (right), with (0.50, 0.53) in red $\tau = 1$ and 0.95, with (0.86, 0.53) in green $\tau = 1$ and 0.94, and with (0.86, 0.89) in dashed green $\tau = 0.84$ and 0.91.

The starting date of the simulations is crucial in terms of synchronisation between power demand and supply. Consequently, it affects the pair-values of τ and required storage. In terms of weather and climate, we can distinguish three periods in the year. The first starts on January 1st (low outside temperature T_o, mainly wind-driven power production), the second on April 1st (higher T_o, solar-driven power production), the third on September 1st (intermediate T_o, comparable intensity between wind and solar power flows, see Fig. 2). To evaluate the effect of the starting date of

the simulation on the results, we reproduced scenario $A.1$ for three starting dates that are representative of the three climatic periods, see Fig. 8 for January and April, and Fig. 4 and Fig. 5 for September.
In the first case (January 1st), the low outside air temperature $T_{\rm o}$ generates high power demand at the beginning of the simulated period, leading to high power demand from the grid and rapid achievement of the authorized threshold of energy use from the grid ($E_{\rm grid}$). During spring, the progressive increase of outside air temperature allows meeting the target air temperature while building up energy storage, which remains largely unused. This storage allows to have all the energy necessary to face the cold season. The opening ratio τ is then found to be maintained at a minimum of 80% even with 100% VRE contributions. In the second case (April 1st), the high temperatures present from the beginning of the simulation allow an immediate satisfaction of the power demand, whatever the energy mix, but also forces an huge seasonal storage which allows to maintain the conditions of growth more constrained during the cold period, very distant in time. The opening ratio τ is found to be even higher than in previous case, with a minimum of 90% with $N = 1$.
Thus the first two cases allow very high VRE penetration rates, *i.e.* with a low impact in terms of opening ratio, which is allowed thanks to a huge storage volume, approximately $E_{\rm ref}/2$, for a duration equivalent to that of the simulation, masking the deep mismatch between production and demand. Since we aim at describing the impact of VRE penetration in production, we impose as an operating condition to keep storage at the lowest possible level. This is achieved by starting the simulation in late summer *i.e.* September 1st, which is the starting date shown in the results. In this case, the wind generation allows to simultaneously satisfy the reduced demand thanks to the mitigated temperatures and to provide the seasonal storage, which will be of smaller amplitude and shorter term than in the two previous cases, since the period of greatest energy consumption ideally starts slightly after the period of greatest wind-power production.

However, energy loss over transportation of electricity increases with distances, thus the larger the network the greater the required energy production compared to the case of solar panels and wind turbines collocated with energy uses.

Effective power production and efficiencies

We have represented figure 9 the power effectively produced by a square meter of solar panel and by a square meter of wind turbine. To obtain the power produced by a wind turbine, it is advisable to multiply the surface power by the area swept by the blades of the turbine, typically $5\,000\,\mathrm{m}^2$ for a wind turbine of 80 m. We can see that these are closely correlated

to the solar power flux and wind speed, shown in figure 2. The efficiency of the conversion systems is adapted to their environment. In the case of wind turbines, the maximum efficiency is obtained for wind speeds between 6 and 12 m/s, which represents most of the winter season. In this case, the efficiency appears approximately constant, and the power produced depends only on the wind speed.

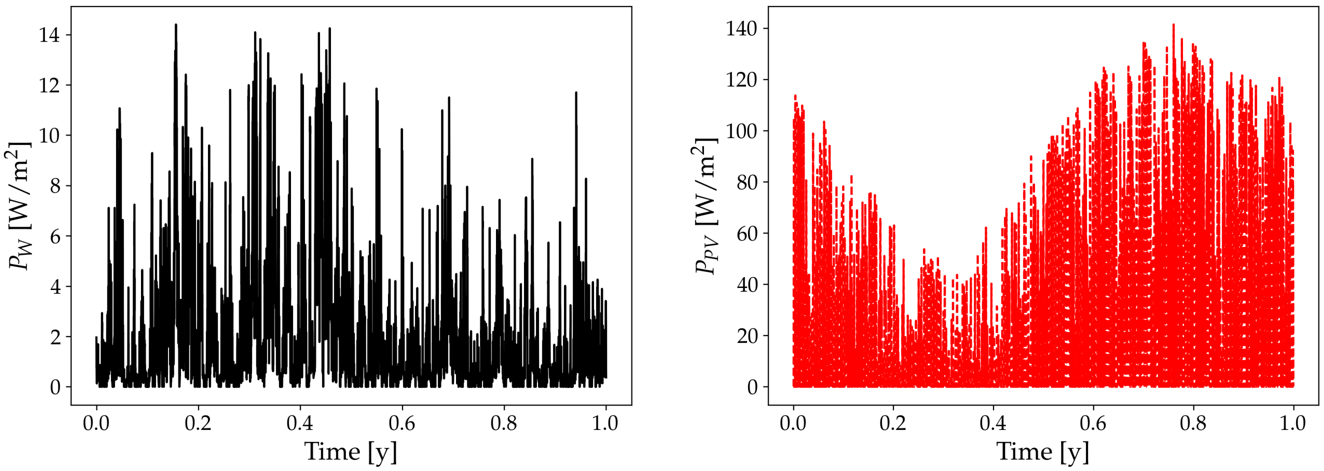

Fig 9. Annual effective power production. Left the wind power produced per square meter of wind turbine. The surface considered here is the one covered by the blades of the blade, typically 5 000 m^2 for a 80 m wind turbine. Right is the PV power produced per square meter of PV panel. The corresponding incident wind velocity and solar power flux are shown in figure 2.
